# Modification of Early Response of *Vitis vinifera* to Pathogens Relating to Esca Disease and Biocontrol Agent Vintec^®^ Revealed By Untargeted Metabolomics on Woody Tissues

**DOI:** 10.3389/fmicb.2022.835463

**Published:** 2022-03-02

**Authors:** Justine Chervin, Ana Romeo-Oliván, Sylvie Fournier, Virginie Puech-Pages, Bernard Dumas, Alban Jacques, Guillaume Marti

**Affiliations:** ^1^Laboratoire de Recherche en Sciences Végétales, Université de Toulouse, CNRS, UPS, Toulouse INP, Toulouse, France; ^2^Metatoul-AgromiX Platform, LRSV, Université de Toulouse, CNRS, UPS, Toulouse INP, Toulouse, France; ^3^MetaboHUB-MetaToul, National Infrastructure of Metabolomics and Fluxomics, Toulouse, France; ^4^Unité de Recherche Physiologie, Pathologie, et Génétique Végétales (PPGV), INP PURPAN, Université de Toulouse, Toulouse, France

**Keywords:** *Vitis vinifera*, Esca disease, metabolomics, biomarkers, biocontrol

## Abstract

Esca disease is one of the most destructive grapevine trunk diseases. *Phaeoacremonium minimum* and *Phaeomoniella chlamydospora* are two of the known fungal pathogens associated with this disease. Today, biocontrol agents against Esca are mainly based on the use of the strain of the mycoparasite fungal genus *Trichoderma* such as the Vintec^®^ product. The aim of this study was to investigate early response of woody tissues to Esca pathogens and identify metabolites that could be correlated with a biocontrol activity within a complex woody matrix. An untargeted liquid chromatography–high-resolution mass spectrometry metabolomic approach coupled to a spectral similarity network was used to highlight clusters of compounds associated with the plant response to pathogens and biocontrol. Dereplication highlighted the possible role of glycerophospholipids and polyphenol compounds, the latest mainly belonging to stilbenoids. Antifungal activity of some relevant biomarkers, evaluated *in vitro* on *Phaeomoniella chlamydospora* and *Botrytis cinerea*, suggests that some of these compounds can play a role to limit the development of Esca pathogens in planta.

## Introduction

Grapevine trunk diseases (GTDs) are of growing concern among worldwide viticulture. These diseases concern different organs of the plant, some causing the progressive death of vines in vineyards. These losses influence the productivity of the vineyard and, therefore, represent a considerable economic impact in the viticultural sector (Hofstetter et al., 2012; Fontaine et al., 2016). Grapevine trunk diseases are associated with the presence of different pathogenic fungal species, which can affect vines at different stages of their life cycle (Bertsch et al., 2013; Bruez et al., 2014).

Esca is one of the most complex and destructive trunk diseases. The Esca disease comprises different syndromes, depending on the symptoms, the age of the plant, and the fungal species associated (Graniti et al., 2000; Surico, 2009; Bertsch et al., 2013). One of the syndromes, named Esca, is associated with white rot in the trunk, and it is caused by fungal species of the genus *Formitiporia* (Bruno and Sparapano, 2007). The three vascular syndromes—brown wood streaking (Mugnai et al., 1999), Petri disease, and grapevine leaf stripe disease (GLSD) (Bertsch et al., 2013)—are associated with *Phaeomoniella chlamydospora* (*P. chlamydospora*), *Phaeoacremonium minimum* (*P. minimum*), and *other Phaeoacremonium* ssp. (Luque et al., 2009; Úrbez-Torres et al., 2014). The presence of these pathogens in the wood affects not only the trunk but also leaves and berries. Symptoms on leaves start with chlorotic areas becoming necrotic and conferring a “tiger striped” appearance. On berries, dark purple spots on the epidermis can be observed (Mugnai et al., 1999). The last syndrome, Esca proper, corresponds to the co-occurrence of Esca and GLSD in the same plant (Surico, 2009).

In the absence of curative methods, since the banishment of sodium arsenite, there are numerous strategies to limit the occurrence of GTDs both in nurseries and in the field (Mondello et al., 2018a,b). One of these strategies includes the use of biocontrol agents (BCAs). Biocontrol agents represent promising alternatives to conventional phytosanitary products (Ram et al., 2018). *Trichoderma* spp. have been largely studied and are of great interest as biocontrol agents (Moya et al., 2020; Poveda, 2021). In viticulture, there exist two *Trichoderma-*based commercial solutions to control GTDs: Vintec^®^ (Belchim Crop Protection) and Esquive^®^ (Agrauxine by Lesaffre). Some microorganisms have also achieved good results in grapevine protection against GTDs, such as the oomycete *Pythium oligandrum* (Yacoub et al., 2016) or a number of bacteria (Trotel-Aziz et al., 2019; Haidar et al., 2020). *Pythium oligandrum* is known to have an antifungal effect against different plant fungal pathogens (Gerbore et al., 2014). Concerning GTDs, the presence of this oomycete reduces *P. chlamydospora* infection (Yacoub et al., 2016). The known modes of action of *P. oligandrum* are diverse and, as well as *Trichoderma* spp., it can enhance plant defense responses (Yacoub et al., 2020). In the case of bacteria, Trotel-Aziz et al. (2019) found that *Bacillus subtilis* protects grapevine against *Neofusicoccum parvum*, the causal agent of Botryosphaeria dieback, by the activation of detoxification process in the plant. In addition, Haidar et al. (2021) found that bacteria from the genus *Paenibacillus* produced antifungal metabolites and volatile organic compounds that reduced the infection of *N. parvum* in the grapevine’s trunk.

*Trichoderma* species have been widely studied for this purpose. Their ability to protect plants resides in the combination of several factors: (i) the enhancement of nutrient uptake and mobilization, (ii) mycoparasitism and competition with the pathogens, and (iii) induction of plant systemic resistance (Vinale et al., 2008; Braun et al., 2018). Vintec^®^, a formulation of *Trichoderma atroviridae* SC1 (TASC1), is one of the main approved BCAs today to fight trunk diseases (Esca, black dead arm, eutypiose). Its efficiency has been successfully tested in both nursery and vineyard (Berbegal et al., 2020).

In order to better understand this disease, different studies were focused on the physiological modifications on Esca-affected vines (Fontaine et al., 2016). Early studies reported the accumulation of stress metabolites in the wood of Esca-diseased vines (Amalfitano et al., 2000, 2011). Modifications in phenolic compounds in roots showing an early decline were also observed (Del Rio et al., 2001). However, most available works are based on targeted approaches linked with polyphenol compound changes as described in Amalfitano et al. (2011); Lima et al. (2012), or Rusjan et al. (2017), for example.

Untargeted approaches as transcriptomics and metabolomics are of prime interest to study the overall modifications induced in plants during disease outcomes. Recently, several omic studies were assessed in the context of Botryosphaeria dieback issue. A metabolomic study based on wood response with the pathosystem *Neofusicoccum parvum* revealed significant modifications of primary and secondary metabolites after inoculation, in particular a decrease in sugars and an increase in stilbenes and flavonoids (Labois et al., 2020). Another study also reported the importance of lipids in understanding this disease and showed that in diseased vines, phytochemicals, in particular stilbenes and flavonoids, mainly characterized the brown stripe area, whereas lipids, in particular phospholipids, mainly characterized the adjacent white area (Lemaitre-Guillier et al., 2020).

Regarding the Esca issue, a first report on the pathosystem *Vitis vinifera*/Esca upon untargeted metabolomics through nuclear magnetic resonance spectroscopy-based metabolomic showed that a double stress induced by fungi and drought led to the change of several amino acids (Lima et al., 2017b). Nowadays, the majority of studies focus on leaf metabolomes such as the investigation of phenolic modification in leaves of *Vitis vinifera* cv. Alvarinho revealing an apparent accumulation of phenolic compounds in diseased leaves (Lima et al., 2017a). Another work focusing on alterations in leaf metabolome reported that changes, especially concerning phenylpropanoids but also carbohydrates and amino acids, could already be observed prior to symptom appearance (Magnin-Robert et al., 2017). Lipidomic analysis of leaves of Esca-affected grapevine was also conducted (Goufo and Cortez, 2020) and notably showed a general increase in most lipids that correlated with symptom progression.

The present study investigates the response of grapevine woody tissue to fungal infection with the biocontrol agent (BCA) Vintec^®^ (IV), a mixture of both fungi *P. chlamydospora* and *P. minimum* (IPP), as it is usually observed in a natural context, and their combination (IVPP), at an early stage of infection by an ultra-high-performance liquid chromatography–high-resolution mass spectrometry (UHPLC-HRMS)-based metabolomic approach.

The aim of this UHPLC-HRMS approach is to highlight metabolite changes that could be further considered as early biomarkers of infection. This is crucial since fungi associated with Esca disease are known to colonize grapevines during several years without any detectable symptoms in aerial organs like leaves (Bruez et al., 2016).

For this study, a pathosystem was set up to mimic the natural events by co-inoculation of pathogens with and without a biocontrol agent in the trunk of canes. The aim is to be as close as possible to the vineyard to propose biomarkers to monitor both Esca pre-emergence and biocontrol effects.

## Materials and Methods

### Standards

Individual solutions of natural products (NPs) compounds from Metasci (Metasci, Toronto, Canada) and Chemfaces (Wuhan, Hubei) were prepared at 100 μg/ml according to suppliers’ recommendations. Belchim Crop Protection (Lissieu, France) supplied the BCA Vintec^®^.

### Fungal Material

*Phaeoacremonium minimum* (CBS 100398), *Phaeomoniella chlamydospora* (CBS 239.74), and *Botrytis cinerea* (CBS 131.28) strains came from the fungal biodiversity center (Centraalbureau voor Schimmelcultures, CBS. Utrecht, the Netherlands). Fungal isolates were grown in individual Petri plates containing the malt extract-agar (MEA) medium for five weeks at 26°C in obscurity before use.

### Plant Culture

One-year-old canes of *Vitis vinifera* L. cv Cabernet-Sauvignon clone 15 purchased from a French nursery (Pépinières Daydé, Montans, Midi-Pyrénées, France) of the 2019 and 2021 seasons were divided in cuttings containing two dormant nodes. Cuttings were then kept at 5°C for one night. Before planting, their surface was disinfected. At first, cuttings were soaked for 30 s in a 10-L water bath with 5-ml bleach (2.6% active chlorine) and then rinsed twice with 10 L of tap water. Then, cuttings were stored overnight at 4°C in a 10-L water bath containing 5-ml 8-hydroxyquinoline sulfate (0.05% Cryptonol^®^, Chauvin, France), rinsed three times with 10 L of demineralized water. They were planted in moist autoclaved (121°C, 15 min) rock wool to allow disbudding and rooting. At this point, plants were watered twice a week. Four weeks later, cuttings were transferred from the rock wool into individual pots containing 100 g of autoclaved soil (PAM2, PROVEEN Substrates). Plants were finally grown in a plant growth chamber at 25°C, with 45% of humidity, a photoperiod of 12 h/12 h, and watered three times weekly.

### Plant Inoculation

The following conditions were studied, and for each, 16 plants were used: Injured/Not inoculated (INi); Injured/Vintec^®^ (IV); Injured/*P. minimum* + *P. chlamydospora* (IPP); Injured/Vintec^®^ + *P. minimum* + *P. chlamydospora* (IVPP).

The injured plants were drilled at the internode, as described in Reis et al. (2019). Plants injured but not inoculated with Vintec^®^ received 20 μl in the hole of sterile distilled water; plants injured and inoculated with the biocontrol received 20 μl in the hole of a suspension of the product Vintec^®^ at 2 g L^–1^ of formulated product 5 days before infection with Esca fungi (T-5d). The injury was covered with a strip of Parafilm (American National Can, Chicago, IL).

Inoculation with fungi *P. minimum* and *P. chlamydospora* at T0 was performed by drilling again all the plants at the same spot, and a plug of agar-agar colonized by fungi was directly introduced. All the plants were grown in a plant growth chamber at 25°C, with 45% of humidity and a photoperiod of 12 h/12 h for 3 or 6 weeks.

### Wood Sample Extraction for Metabolomic Analysis and Fungal Colonization Quantification

For metabolomic analysis, samples were harvested at 3 weeks (T3) and 6 weeks (T6) after treatment. We performed an analysis on samples collected above and behind the inoculation site, and these preliminary data did not show a systemic response. This observation agreed with Pierron et al. (2015), showing that wood responses to *P. minimum* (*P. aleophilum*) were mainly restricted to the inoculation point six weeks post-infection. Thus, around 1 cm of wood in the inoculation part (I) was collected and immediately dipped in liquid nitrogen. Likewise, wood samples of the same treatment were pooled in 6 tubes containing 3 pieces of wood from 3 different plants. Each sample was then grounded with a Mixer Mill MM 300 (Retsh, Eragny sur Oise, France) by applying 28 oscillations per second for 1 min. Around 100 mg of powder was then placed in a FastPrep tube (MP Biomedicals Lysing Matrix D, Illkirch, France) and kept at −80°C.

For sample extraction, 1 ml of 70% EtOH was added per 100 mg followed by three cycles of 20 s at 6 m/s in the FastPrep while keeping samples in ice between each cycle. After a centrifugation at 4°C and 12,000 rpm for 10 min, the supernatant was collected and a second extraction was achieved on the residue. The two supernatants were pooled, transferred to vials, and kept at −20°C before injection. An extraction blank (without plant material) and quality control (QC) samples were also prepared for extraction and analytical validation.

A set of three plants of each treatment was reserved for evaluating fungal colonization six weeks post-inoculation via qPCR quantifications. DNA extraction was performed using a CTAB/CIA-based protocol combined with a Qiagen DNA extraction kit (DNeasy plant mini Kit, Qiagen, United States), as described in Romeo-Oliván et al. (2021). qPCR reactions were conducted with the GoTaq^®^ qPCR System (Promega) and ABI 7500 Real-Time PCR Cycler (Applied Biosystems, Foster City, United States) following this cycling program: 15 min at 95°C (denaturation), 40 cycles of 15 s at 95°C and 45 s at 62°C (annealing and extension), and 40 min from 60 to 95°C (melting analysis). Specific primers targeting the β-tubulin gene of *P. chlamydospora* and *P. minimum* were previously described (Pouzoulet et al., 2013) as well as those amplifying the endochitinase 42 gene of *T. atroviridae SC1* for Vintec^®^ (Savazzini et al., 2008). They were used at 0.5 μM in a final reaction volume of 10 μl. The number of gene copies of *P. chlamydospora* and *P. minimum* was estimated using a standard curve (Pouzoulet et al., 2013). Two-way ANOVA and Dunnett’s *post hoc* test (p ≤ 0.05) were used to compare the gene copy number of the various modalities using GraphPad Prism 8 version 8.3.0 (San Diego, California).

For Vintec^®^, we only considered the presence/absence of amplification.

### Ultra-High-Performance Liquid Chromatography–High-Resolution Mass Spectrometry Profiling

Ultra-high-performance liquid chromatography-high-resolution MS (UHPLC-HRMS) analyses were performed on a Q Exactive Plus quadrupole (Orbitrap) mass spectrometer, equipped with a heated electrospray probe (HESI II) coupled to a U-HPLC Ultimate 3000 RSLC system (Thermo Fisher Scientific, Hemel Hempstead, United Kigdom). Separation was done on a Luna Omega Polar C18 column (150 mm × 2.1 mm i.d., 1.6 μm, Phenomenex, Sartrouville, France) equipped with a guard column. The mobile phase A (MPA) was water with 0.05% formic acid (FA), and the mobile phase B (MPB) was acetonitrile with 0.05% FA. The solvent gradient was 0 min, 100% MPA; 1 min, 100% MPA; 22 min, 100% MPB; 25 min, 100% MPB; 25.5 min, 100% MPA; and 28 min, 100% MPA. The flow rate was 0.3 ml/min, the column temperature was set to 40°C, the autosampler temperature was set to 5°C, and the injection volume was fixed to 5 μl. Mass detection was performed in positive ionization (PI) mode at resolution 35,000 power [full width at half-maximum (fwhm) at 400 m/z] for MS1 and 17,500 for MS2 with an automatic gain control (AGC) target of 1 × 10^6^ for full scan MS1 and 1 × 10^5^ for MS2. Ionization spray voltages were set to 3.5 kV, and the capillary temperature was kept at 256°C. The mass scanning range was m/z 100-1500. Each full MS scan was followed by data-dependent acquisition of MS/MS spectra for the six most intense ions using stepped normalized collision energy of 20, 40, and 60 eV.

### Data Processing

Ultra-high-performance liquid chromatography–high-resolution mass spectrometry (UHPLC-HRMS) raw data were processed with MS-DIAL version 4.70 (Tsugawa et al., 2015) for mass signal extraction between 100 and 1,500 Da from 0.5 to 18.5 min. Respectively, MS1 and MS2 tolerance were set to 0.01 and 0.05 Da in the centroid mode. The optimized detection threshold was set to 1.5 × 10^6^ concerning MS1 and 10 for MS2. Peaks were aligned on a QC reference file with a retention time tolerance of 0.1 min and a mass tolerance of 0.015 Da. Peak annotation was performed with an in-house database built on an MS-FINDER model (Tsugawa et al., 2016).

MS-DIAL data were then cleaned with the MS-CleanR workflow (Fraisier-Vannier et al., 2020) by selecting all filters with a minimum blank ratio set to 0.8, a maximum relative standard deviation (RSD) set to 40, and a relative mass defect (RMD) ranging from 50 to 3,000. The maximum mass difference for feature relationships detection was set to 0.005 Da and the maximum RT difference to 0.025 min. The Pearson correlation links were considered with correlation ≥ 0.8 and statistically significant with α = 0.05. Two peaks were kept in each cluster with the most intense and the most connected. The kept features (*m/z* × RT pairs) were annotated with MS-FINDER version 3.52. The MS1 and MS2 tolerances were, respectively, set to 5 and 15 ppm. Formula finders were only processed with C, H, O, N, P, and S atoms. Databases (DBs) based on *Vitis* (genus), Vitaceae (family), *Trichoderma* (genus of the BCA strain), and Togniniaceae (the family of the two pathogenic fungi) were constituted with the dictionary of natural product (DNP, CRC press, DNP on DVD v. 28.2). The internal generic DBs from MS-FINDER used were KNApSAcK, PlantCyc, HMDB, LipidMaps, NANPDB, and UNPD. Annotation prioritization was done by ranking *Vitis* DB, followed by Vitaceae DB, *Trichoderma* and Togniniaceae DBs, and finally generic DBs using the final MS-CleanR step.

### Statistical Analysis

Statistical analyses were made with Orange 3.30.1 (Demsar et al., 2013). All data were scaled by unit variance scaling (UV) before multivariate analysis. ANOVA models were proposed to rank features and highlight biomarkers of plant response to the various studied treatments. Models confronting only two treatments at once were built taking as reference the sample Injured/Not inoculated (INi). A random forest model was used to validate the comparisons based on the cross-validation algorithm. Two-way ANOVA and Dunnett’s *post hoc* test (*p* ≤ 0.05) were used to check biomarker significance for both kinetics in comparison to references INi with GraphPad Prism 8 version 8.3.0 (San Diego, California).

The heatmap of revealed biomarkers based on average peak area was built with the web-interface MetaboAnalyst version 4.0 (Chong et al., 2019).

### Mass Spectral Similarity Network

The.msp PI and metadata files generated at the end of the MS-CleanR workflow were imported into MetGem version 1.2.2. A mass spectral similarity network was built with a cosine score cutoff fixed at 0.6, a maximum of ten connections between nodes, and at least four matched peaks. The resulting network was imported into Cytoscape12 version 3.7.2 to tune visualization. Node color was based on chemical classes, and the size of revealed biomarkers was increased. The edge width was deepened according to the cosine score.

### *In vitro* Bioassays

Antifungal activity of pure available standard metabolites was investigated *in vitro* in 96-well plate cultures by measuring fungal growth. Standard solutions were previously dissolved in 8% DMSO in water with concentration ranging from 0.1 to 1.6 mg/ml. Three final concentrations in each well were investigated for each standard, based on antifungal activity results found in the literature: 100, 10, and 1 μg/ml for α-viniferin (Langcake and Pryce, 1977); 50, 5, and 0.5 μg/ml for viniferin (Langcake, 1981), and finally 25, 2.5, and 0.25 μg/ml for pterostilbene (Jeandet et al., 2002). As no information was found on oxyresveratrol, a higher range of concentration was studied: 400, 40, and 4 μg/ml.

To obtain *Botrytis cinerea* and *P. chlamydospora* spores, two plugs of agar colonized by the mycelium of each were cut, placed in 1-ml sterile water, and vortexed. The spore concentration was adjusted under a microscope using a Malassez counting chamber to 10^3^ spores/ml with water.

Triplicates using *Botrytis cinerea* spores as the positive control as well as *P. chlamydospora* spores as the target pathogen were prepared for each standard concentration. To the well, 130 μl of malt extract medium (0.01 g/ml) was added, followed by 20 μl of the spore suspension and 50 μl of standard solutions. For the negative control, 20 μl of the spore suspension was replaced by 20 μl of sterile distilled water. Plates were then incubated at 26°C for three days. The absorbance was measured at 600 nm in a microplate reader (Infinite 200 Pro Nano, Tecan, Austria). IC_50_ (concentrations causing an inhibition of 50% of fungal growth) were then calculated using GraphPad Prism 8 version 8.3.0 (San Diego, California). Control replicates were prepared to subtract the compound absorbance.

## Results

### Fungal Inoculation on *Vitis vinifera*

**FIGURE 1:**
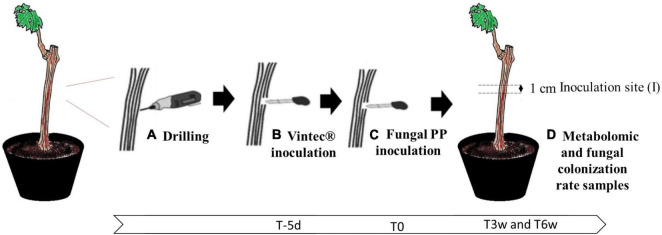
Grapevine model used to study Esca disease in laboratory conditions: **(A)** drilling injury site, **(B)** inoculation of biocontrol agent (BCA) Vintec^®^ or sterile water, 5 days before fungal PP inoculation (T-5d), **(C)** fungal PP inoculation (T0), and **(D)** sampling site for metabolomic study after 0, 3, and 6 culture weeks (T0, T3, and T6) or fungal colonization rate (T6).

The dataset was built on a reductionist model to study the local effect of the BCA Vintec^®^ in the metabolic regulation of a plant challenged or not with the two fungal pathogens and highlight early biomarkers of pathogens and biocontrol presence (Figure 1).

**FIGURE 2:**
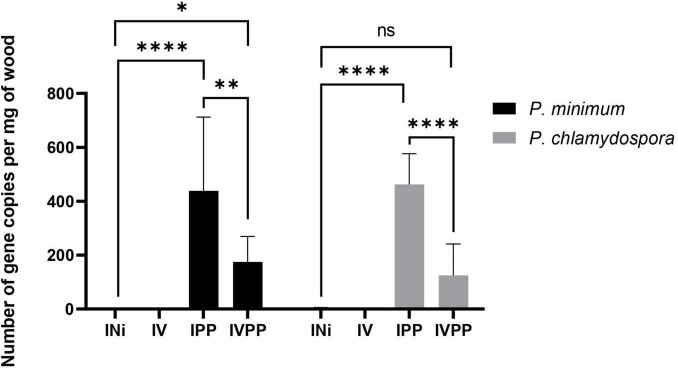
Gene copy number per mg of wood of *Phaeoacremonium minimum* (black) and *Phaeomoniella chlamydospora* (gray) 6 weeks post-infection (T6w). INi = control, injured samples; IV = samples inoculated with Vintec^®^; IPP = samples infected with *P. chlamydospora* and *P. minimum*; IVPP = samples pre-inoculated with Vintec^®^ and infected with *P. chlamydospora* and *P. minimum*. *N* = 3. * Significance: 0.1234 (ns), 0.0332(*), 0.0021(^**^), < 0.0001(^****^).

*Phaeomoniella chlamydospora* and *P. minimum* colonization was estimated via quantifications by qPCR of their β-tubulin gene (Figure 2). The results indicated that the wood was still colonized six weeks post-infection by the two fungal pathogens in IPP and IVPP samples. Moreover, a significant reduction of colonization by *P. chlamydospora* and *P. minimum* due to the BCA Vintec^®^ was observed. As expected, control samples (INi) and samples inoculated with Vintec^®^ (IV) were free of pathogenic DNA. The presence of *T. atroviridae SC1* for Vintec^®^ pretreatment was also assessed via qPCR, confirming the colonization by *T. atroviridae* in the biocontrol-treated IV and IVPP samples (Supplementary Table 1). Altogether, these results corroborated that the data obtained further are related to the presence of either the pathogens, the BCA, or the combination of the three.

### Metabolomic Analysis of Wood Samples

Ultra-high-performance liquid chromatography–high-resolution mass spectrometry (UHPLC-HRMS) profiles of all the extracts afforded 239 features (*m/z*-RT pairs) in the PI mode after application of the MS-CleanR workflow. Among these features, 85% of them were annotated using different DBs: 3% (*Trichoderma)*, 1% (Togniniaceae), 4% (Vitaceae), 42% (*Vitis*), and 35% (generic internal MS-FINDER DBs). Fungal metabolites were found in samples inoculated, and their intensity was between 5 × 10^5^ and 3 × 10^6^. As the background threshold was estimated to 1.5 × 10^6^, they appeared in the minority not only in terms of number but also in terms of intensity. Thus, separation of samples in principal component analysis (PCA) was mainly due to plant metabolites, and we only focused on them in the rest of the paper.

**FIGURE 3:**
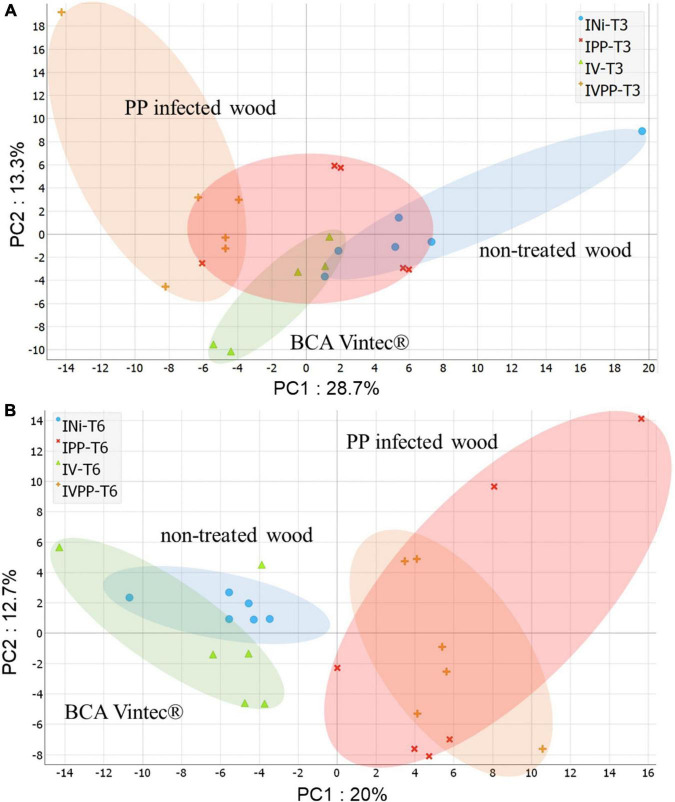
PCA score plot of ESI-PI data from wood extract of Vitis cuttings of kinetics T3 **(A)** and kinetics T6 **(B)** (in color). Colored circles arbitrarily enclose treatment types: Injured/Not inoculated (INi) samples; Injured/Vintec^®^ (IV); Injured/*P. chlamydospora* + *P. minimum* (IPP) and Injured/*P. chlamydospora* + *P. minimum* + Vintec^®^ (IVPP).

PCA score plots of respective kinetics T3 (Figure 3A) and T6 (Figure 3B), providing an unsupervised overview of UHPLC-HRMS fingerprints, marked sample-to-sample variability particularly for PP-infected wood at T3 and T6. It, respectively, displayed 42% and 32.7% of total explained variance using the first two principal components. Quality control samples of the whole dataset (Supplementary Figure 1) were centered on the PCA plot, demonstrating the high reproducibility of the analysis.

More generally, a separation of samples can be seen according to treatment type. Non-treated wood composed of INi class and wood infected with fungi composed of IPP and IVPP classes are separated by PC1 in both kinetics. Interestingly, wood samples only treated with Vintec^®^ are separated by PC1 for kinetics T3 and by PC2 for kinetics T6.

**FIGURE 4:**
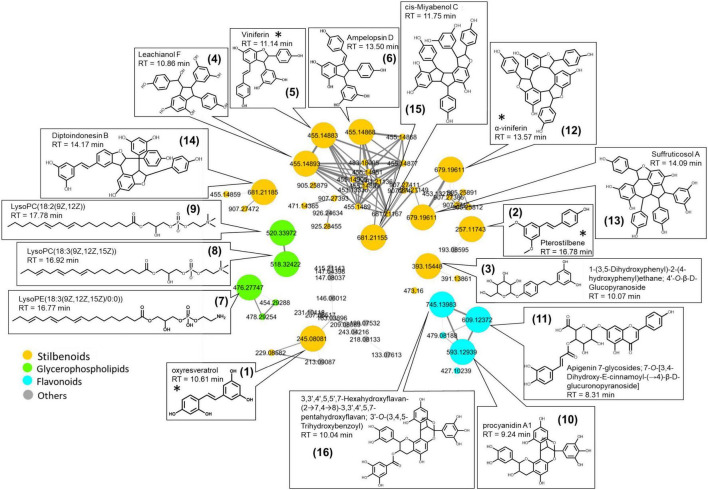
Mass spectral similarity network based on the ESI-PI dataset of ultra-high-performance liquid chromatography–high-resolution mass spectrometry (UHPLC-HRMS) performed on wood extracts that highlights the characteristic biomarkers in response to wood treatment. Color tag is based on chemical class determined with ClassyFire. Putative structures were based on HRMS and MS/MS spectra. * Identification confirmed by analytical standards.

Using the MS-CleanR workflow (Fraisier-Vannier et al., 2020), a cleaner mass spectral similarity network was built to highlight common chemical classes related to plant response to pathogens and Vintec^®^ (Figure 4). Potential biomarkers were revealed by ANOVA models to obtain a classification of the features (*m/z-*RT pairs) for each treatment in comparison with the reference.

All the two-to-two comparisons models were validated by random forest models based on the ROC curve coupled with the area under the curve (AUC). We observed a high AUC score around 0.9 (Supplementary Table 2) indicating a good prediction ability.

Sixteen significant biomarkers correlated to the highest ANOVA coefficient scores were tentatively annotated by interrogating simultaneously *Vitis*, Vitaceae, *Trichoderma*, Togniniaceae, and local databases of natural products in MS-FINDER (Table 1). Three main biomarker clusters were observed, and annotation results gathered from the aligned peak list of LCMS features allowed the highlight of three main chemical classes: stilbenoid, flavonoid, and glycerophospholipids. The molecular formulae of these compounds matched structures already identified in the *Vitis* genus, except the three glycerophospholipids found in generic databases. No biomarkers were annotated with fungal databases.

**TABLE 1:** Summary of annotated compounds most differentially present in the tested conditions.

N°	m/z	RT (min)	Molecular Formula	Error (ppm)	Chemical class^a^	Putative annotation
1	245.08081	10.608	C_14_H_12_O_4_	4.5781	Stilbenoid	Oxyresveratrol^b^
2	257.11743	16.784	C_16_H_16_O_3_	3.4537	Stilbenoid	Pterostilbene^b^
3	393.15448	10.065	C_20_H_24_O_8_	2.5715	Stilbenoid	1-(3,5-Dihydroxyphenyl)-2-(4-hydroxyphenyl)ethane; 4′-*O*-β-D-glucopyranoside^c^
4	455.14893	10.863	C_28_H_20_O_5_	2.3772	Stilbenoid	Leachianol F^c^
5	455.14883	11.139	C_28_H_22_O_6_	2.5969	Stilbenoid	Viniferin^b^
6	455.14868	13.502	C_28_H_22_O_6_	2.9265	Stilbenoid	Ampelopsin D^c^
7	476.27747	16.766	C_30_H_37_NO_4_	4.2820	Glycerophospholipids	LysoPE[18:3(9Z,12Z,15Z)/0:0]^d^
8	518.32422	16.922	C_26_H_48_NO_7_P	1.9158	Glycerophospholipids	LysoPC [18:3(9Z,12Z,15Z)] ^d^
9	520.33972	17.781	C_26_H_50_NO_7_P	2.1966	Glycerophospholipids	LysoPC [18:2(9Z,12Z)] ^d^
10	593.12939	9.237	C_30_H_24_O_13_	1.1363	Flavonoid	Procyanidin A1^c^
11	609.12372	8.31	C_30_H_24_O_14_	2.0669	Flavonoid	Apigenin 7-glycosides; 7-*O*-[3,4-Dihydroxy-E-cinnamoyl-(→4)-β-D-glucuronopyranoside]^c^
12	679.19611	13.571	C_42_H_30_O_9_	1.8345	Stilbenoid	α-viniferin^b^
13	679.19611	14.085	C_42_H_30_O_9_	1.8345	Stilbenoid	Suffruticosol A^c^
14	681.21185	14.168	C_42_H_32_O_9_	0.0866	Stilbenoid	Diptoindonesin B^c^
15	681.21155	11.753	C_42_H_32_O_9_	0.5270	Stilbenoid	cis-Miyabenol C^c^
16	745.13983	10.039	C_37_H_28_O_17_	1.6010	Flavonoid	3,3′,4′,5,5′,7-Hexahydroxyflavan-(2→7,4→8)-3,3′,4′,5,7-pentahydroxyflavan; 3′-*O*-(3,4,5-Trihydroxybenzoyl)^c^

*^a^Determined with ClassyFire (Djoumbou Feunang et al., 2016).*

*^b^Compounds confirmed by injection of standard in the same analytical conditions.*

*^c^Putative annotation based on experimental HRMS, MS/MS, and in silico fragmentation matches restricting interrogation to Vitis genus and Vitaceae family databases.*

*^d^Putative annotation based on experimental HRMS, MS/MS, and in silico fragmentation matches by interrogation of natural product databases within the MS-FINDER software.*

The majority of the biomarkers belong to the stilbenoid class. They are resveratrol monomers (compounds 1, 2, and 3), dimers (compounds 4, 5, and 6), and trimers (compounds 12, 13, 14, and 15). Four of them displayed the identification level 1 according to the Metabolomics Standards Initiative (MSI) (Sumner et al., 2007) as their retention time, and MS/MS fragments perfectly match with the standards injected in the same conditions as samples (RT similarity score > 850; similarity spectrum score > 800): oxyresveratrol, pterostilbene, α-viniferin, and viniferin. For all other compounds, the identification level 2 was proposed according to the MSI.

The three glycerophospholipids were annotated as lysophosphatidylcholines lysoPC (18:2) (9) and lysoPC (18:3) (8), and as lysophosphatidylethanolamine LysoPE (18:3) (7).

Two flavonoids were annotated as procyanidin derivatives (10 and 16), while the last one was annotated as an apigenin derivative (11).

**FIGURE 5:**
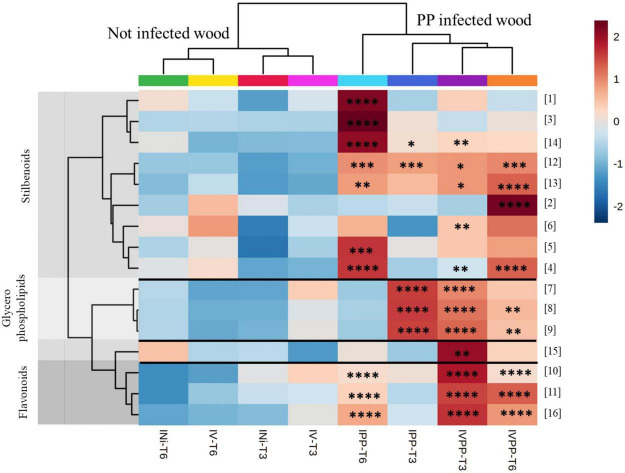
Heatmap of the annotated biomarkers at kinetics 3 weeks (T3) and 6 weeks (T6). The color key is based on the average peak intensity of each feature by class: red color for higher peak intensity and blue color for lower peak intensity. Injured/Not inoculated (INi) samples; Injured/Vintec^®^ (IV); Injured/*P. chlamydospora* + *P. minimum* (IPP) and Injured/*P. chlamydospora* + *P. minimum* + Vintec^®^ (IVPP). Report to Table 1 for metabolite annotation. * Significance in comparison to INi for each kinetics independently [0.0332(*), 0.0021(^**^), 0.0002(^***^), < 0.0001(^****^)].

After tentative annotation, we compared their distribution among treated conditions. On the heatmap representing the average peak intensity by class of each biomarker (Figure 5), it can be seen that these significant features are overproduced in the presence of pathogenic fungi alone (IPP) and in combination with BCA Vintec^®^ (IVPP), validating our approach. In addition, the hierarchical clustering analysis, based on the sample class, separates well the non-infected wood samples, including those with Vintec^®^ alone, from the pathogen-inoculated infected wood samples.

Among the different metabolites, flavonoids are mainly present at kinetics T3 and T6 with IPP and IVPP (metabolites 10, 11, and 16) and glycerophospholipids at kinetics T3 with IPP and IVPP (metabolites 7, 8, and 9). Suffruticosol A (13) and α-viniferin (12), two resveratrol trimers, are overall equally expressed with IPP and IVPP at both kinetics. *Cis*-miyabenol C (15) and ampelopsin D (6) are mainly overproduced with IVPP at kinetics T3, while pterostilbene (2) is mainly overproduced with IVPP at kinetics T6. Oxyresveratrol (1), the bibenzyl derivative (3), and diptoindonesin B (14) are mainly overproduced with IPP at kinetics T6. Finally, all other annotated resveratrol dimers (metabolites 4 and 5) are overproduced with IPP and IVPP at kinetics T6. ANOVA comparisons can be found in Supplementary Figure 2.

Interestingly, all biomarkers overproduced with IVPP were not significantly overproduced in the presence of BCA Vintec^®^ alone, as well as for kinetics T3 and for kinetics T6. Thus, it resulted that Vintec^®^ treatment alone induces a weak grapevine defense response, which seemed rather correlated with plant reaction to pathogenic fungal infection. Nevertheless, some metabolites, in particular oxyresveratrol (1), the bibenzyl derivative (3), and diptoindonesin B (14), were less produced with pathogens in combination with Vintec^®^ than with pathogens alone. Thus, Vintec^®^ seems to reduce some metabolic response to pathogen attack.

**FIGURE 6:**
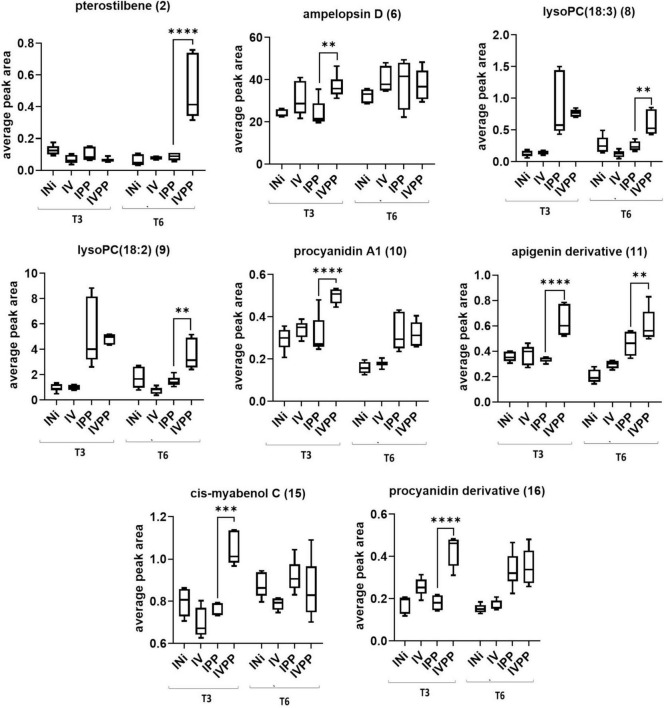
Two-way ANOVA and Dunnett’s *post hoc* test (*p* ≤ 0.05) of metabolites overproduced with the combination Vintec^®^–pathogens. 0.0021(^**^), 0.0002(^***^), < 0.0001(^****^).

From another side, Vintec^®^ increases plant response to pathogen treatment (Figure 6), in particular regarding the expression of stilbenoids pterostilbene (2) at T6 and *cis*-miyabenol C (15) at T3, as well as flavonoids 10, 11, and 16 at T3. To a lesser extent, it also stimulates the production of lysoPCs 8 and 9 at T6 and ampelopsin D at T3.

The reproducibility of the experiment and the raw materials were confirmed for kinetics T3 with canes of *Vitis vinifera* L. cv Cabernet-Sauvignon clone 15 during the 2021 season (data not shown). UHPLC-HRMS analysis revealed a metabolic response quite constant as 11 of the 16 highlighted biomarkers were also overproduced in IPP and IVPP samples comparatively to control or BCA Vintec^®^ treatment of the 2021 season (Supplementary Figure 3).

### Validation of Stilbenoids Antifungal Activity

Antifungal activity of four stilbenoids standards was investigated on *Botrytis cinerea* and *P. chlamydospora* fungal growth. IC_50_ was determined (Table 2), and the results pointed out that all stilbenes showed an antifungal activity on both fungi, *P. chlamydospora* being the more susceptible. Pterostilbene and viniferin had the highest antifungal activities for both fungi, whereas oxyresveratrol presented the lowest activity.

**TABLE 2:** IC_50_ of the standard solutions on *Botrytis cinerea* and *Phaeomoniella chlamydospora* spores 3 days after onset of treatment.

	IC_50_ Botrytis (μg/ml)	IC_50_ Pch (μg/ml)
Oxyresveratrol (1)	162	40.0
Pterostilbene (2)	20.3	7.3
Viniferin (5)	37.5	8.6
α-viniferin (12)	92.8	13.5

## Discussion

The objective of this study was to characterize the grapevine wood metabolome changes, during a 6-week kinetics, after the inoculation of the Esca pathogens *Phaeomoniella chlamydospora* and *Phaeoacremonium minimum*, and of the commercialized BCA Vintec^®^ formulated with *Trichoderma atroviridae* SC1, within the framework of Esca disease. This work was expected to identify some biomarkers of Esca attack that could be used to further evaluate BCA efficiency in vineyard conditions. Even if BCA can exhibit many different modes of action on targeted disease, the objective was to demonstrate the prime interest of metabolomics on the semi-ligneous and quite challenging Esca pathosystem.

From an analytical point of view, the PI mode was preferred as MS/MS spectra were of better quality than in the NI mode, thus improving feature annotation using MS-FINDER and network feature clusterization. In order to highlight significant features based on pathogens and BCA presence, a classification model between treatment and UHPLC-HRMS fingerprints was realized by random forest. In addition, a mass spectral similarity network was built and allowed the distinction of three main significant clusters from three chemical classes: flavonoids, glycerophospholipids, and mainly stilbenoids as resveratrol monomers, dimers, and trimers. The majority of highlighted biomarkers were annotated from a *Vitis* database. Interestingly, none of them were fungal metabolites even if nearly 5% of all features were annotated with fungal databases. Indeed, the peak intensity of these metabolites was lower than the one of plant metabolites, and they were often close to the background noise demonstrating the prevalence of wood metabolites using this untargeted metabolomic approach. However, their presence is a very good indicator of the presence of pathogens as well as BCA. These results can stimulate further investigation to follow the presence/absence of these different partners in the time course of Esca disease, which show a slow decaying evolution. This could be particularly helpful to understand the first steps of this disease especially when there are no symptoms.

Stilbenoids belong to the phenylpropanoid pathway and include stilbenes, bibenzyls, and phenanthrenes derivatives. They are found as constitutive compounds in several plant families especially Vitaceae (Rivière et al., 2012). As mentioned above, they are synthesized through the phenylpropanoid pathway as well as the flavonoids. However, there is an early divergence between these classes at the p-coumaroylCoA level that can enter either the stilbenoid way through the key stilbene synthase or the flavonoids way thanks to chalcone synthase isomerase (Ali et al., 2011). They are widely considered as phytoalexins as they play a protective role in plant defense to fungal pathogen infection, such as the detected pterostilbene in the presence of *Plasmopara viticola* (Langcake et al., 1979; Pezet et al., 2004). These compounds are also of great interest since they exhibit many biological properties, in particular cardioprotection, neuroprotection, antidiabetic properties, depigmentation, anti-inflammation, cancer prevention, and treatment (Akinwumi et al., 2018). Several other particular stilbenoids were previously reported in literature and isolated from diseased grapevine wood: the resveratrol dimers leachianol F as well as the resveratrol trimers α-viniferin and cis-miyabenol C (Amalfitano et al., 2011). It was also previously shown that infection of *Vitis vinifera* with *Phaeomoniella chlamydospora* induced changes in phenolic compounds with a significant increase in their production after this fungal inoculation. The production of viniferin-type stilbenes was, in particular, observed, absent from the control condition (Lima et al., 2012).

The role of stilbenes as plant defense compounds was strengthened by antifungal bioassays on four available standards. Our results on positive control *Botrytis cinerea* support the data from literature. It was previously shown that ED_50_ (effective dose to obtain 50% mortality) of pterostilbene on *B. cinerea* was around 18 to 20 μg/ml (Jeandet et al., 2002). Regarding α-viniferin, an IC_50_ around 97 μg/ml was observed on *B. cinerea* (Langcake and Pryce, 1977). *P. chlamydospora* appeared to be more sensitive than *B. cinerea.* Pterostilbene and viniferin presented the highest antifungal activities for both fungi, whereas oxyresveratrol showed the lowest activity. A detailed analysis of stilbenoids antifungal activity on various fungi involved in wood disease showed a weak sensibility of *P. chlamydospora* and *P. aleophilum*. *Phaeomoniella chlamydospora* growth was inhibited by pterostilbene and two resveratrol tetramers, vitisins A and B, whereas no stilbenoids had an impact on *P. aleophilum* growth (Lambert et al., 2012). Nevertheless, they found that pterostilbene was the most active stilbenoids agreeing with our results. Still, the apparent divergence regarding sensitivity could be explained by the use of different fungal isolates and by the experimental conditions as our analysis was realized on fungal spores and not mycelium. These results support the key role of stilbenoids in the framework of wood disease.

Flavonoids are also a class of compounds known to play a role in plant defense against pathogens and other environmental stresses (Treutter, 2005). They are often constitutive compounds of the plant with a production enhanced by the stress. In *Vitis vinifera*, they are mainly distributed in leaves, stems, and canes (Goufo et al., 2020). In addition, accumulation of flavonoids, namely procyanidin derivatives, was also highlighted in wood parts of Esca-infected grapevines (Rusjan et al., 2017).

The third class of compounds identified was phospholipids. It was also previously reported that phospholipids lysophosphatidylcholines (lysoPCs), in particular lysoPCs C18:2 and C18:3 identified in this study, increase after pathogen inoculation (Cho et al., 2012). Regarding the leaves of Esca-affected grapevine, a lipidomic study also reported a progressive increase in lipids, including lysophospholipids as lysoPCs C18:2 and C18:3, with symptom progression (Goufo and Cortez, 2020). These compounds are enzymatically produced through phospholipase A activity linked to the signaling pathways of jasmonic acid leading to defense induction (Chandra et al., 1996). However, the precise role of lysoPCs in the interaction between wood tissues and wood pathogens needs further investigation. Nevertheless, lipids appeared to be good biomarkers of grapevine disease as a higher accumulation of them was also observed in asymptomatic diseased grapevine (Lemaitre-Guillier et al., 2020).

*Trichoderma* spp. strains are known to reduce infections caused by trunk disease pathogens, such as *Phaeomoniella chlamydospora* (Pertot et al., 2016) and *Phaeoacremonium minimum* (Carro-Huerga et al., 2020) in a preventive way, as we confirmed here at 6 weeks post-inoculation. Different modes of action have been described for *Trichoderma* spp. to explain their capacity to protect plants against different biotic and abiotic stresses. These explain (i) the competition for resources and space, (ii) the production of toxic compounds, and (iii) the stimulation of plant natural defenses. The ability of *Trichoderma* spp. to enhance plant defenses against different organisms has been already described several times in different pathosystems (Perazzolli et al., 2011; Mastouri et al., 2012; Mathys et al., 2012; Hermosa and Rubio, 2013; Banani et al., 2014; Singh et al., 2019). For instance, a recent study of the application on *T. harzianum* on tomato reported a protective effect against root-knot nematode by the overproduction of reactive oxygen species (ROS), secondary metabolites, and defense-related hormones (Yan et al., 2021). It has also been observed that the application of different *Trichoderma* spp. in eggplant resulted in a higher total phenolic content during the infection with *S. sclerotirum* (Pratap Singh et al., 2021).

Metabolomic results revealed that Vintec^®^ composed of a *Trichoderma atroviride* (TASC1) strain induces a weak metabolomic response alone, sufficient enough to differentiate the treated and not treated plants by metabolomics comparison, but therefore does not have plant defense stimulating effect.

Nevertheless, BCA Vintec^®^ seems to have not only an effect on pathogens but also a priming effect after infection with fungi. Indeed, some metabolites were more produced in IPP samples compared to IVPP samples, especially at T6. This observation could indicate a weaker virulence of pathogens in the presence of Vintec^®^ corroborating BCA capacity to compete and interact with these pathogens. This effect appears within a certain period probably due to the time necessary for the sufficient strain development. On the other hand, Vintec^®^ increases plant response to challenge inoculation with a clear stimulation of the phenylpropanoid pathway with increasing amounts of stilbenoid pterostilbene as well as an increase in flavonoids. Thus, these compounds appear to be good BCA indicators as they are still observable after 3 weeks and even 6 weeks after infection.

## Conclusion

A UHPLC-HRMS based metabolomic study was performed and allowed the dereplication of plant defense metabolites in the framework of Esca disease. Comparison with injured reference (INi) was conducted and significant metabolic changes were observed. Sixteen biomarkers were highlighted and annotated based on MS/MS fragmentation patterns. Four of them were clearly identified thanks to the injection of corresponding standards. These compounds belong mainly to the stilbenoid chemical class, as well as flavonoids and glycerophospholipids known to be involved in plant defense. They were mainly accumulated after two wood treatments: inoculation of the pathogenic fungi, *Phaeoacremonium minimum* and *Phaeomoniella chlamydospora*, and in combination with BCA Vintec^®^. One stilbenoid and three flavonoids were highly associated with the synergy between the BCA and the pathogens. Indeed, they were mainly overproduced when Vintec^®^ was in the presence of fungi. Thus, these four compounds appear to be good indicators of BCA effects in field condition.

## Data Availability Statement

The datasets presented in this study can be found in online repositories. The names of the repository/repositories and accession number(s) can be found below: 10.5281/zenodo.5779519.

## Author Contributions

GM, VP-P, SF, BD, and AJ supervised the project and revised the article. JC was in charge of the plant extraction, the analytical workflow, and the data processing and interpretation. AR-O was in charge of the plant culture and treatment and realized the bioassays. JC and AR-O wrote the article. All authors have read and agreed the published version of the manuscript.

## Conflict of Interest

The authors declare that the research was conducted in the absence of any commercial or financial relationships that could be construed as a potential conflict of interest.

## Publisher’s Note

All claims expressed in this article are solely those of the authors and do not necessarily represent those of their affiliated organizations, or those of the publisher, the editors and the reviewers. Any product that may be evaluated in this article, or claim that may be made by its manufacturer, is not guaranteed or endorsed by the publisher.
