# Supplementary figures and images for: Modification of Early Response of Vitis vinifera to Pathogens Relating to Esca Disease and Biocontrol Agent Vintec® Revealed By Untargeted Metabolomics on Woody Tissues
Source: Front Microbiol. 2022 Mar 2;13:835463. doi: 10.3389/fmicb.2022.835463 (PMC8924477; doi:10.3389/fmicb.2022.835463)

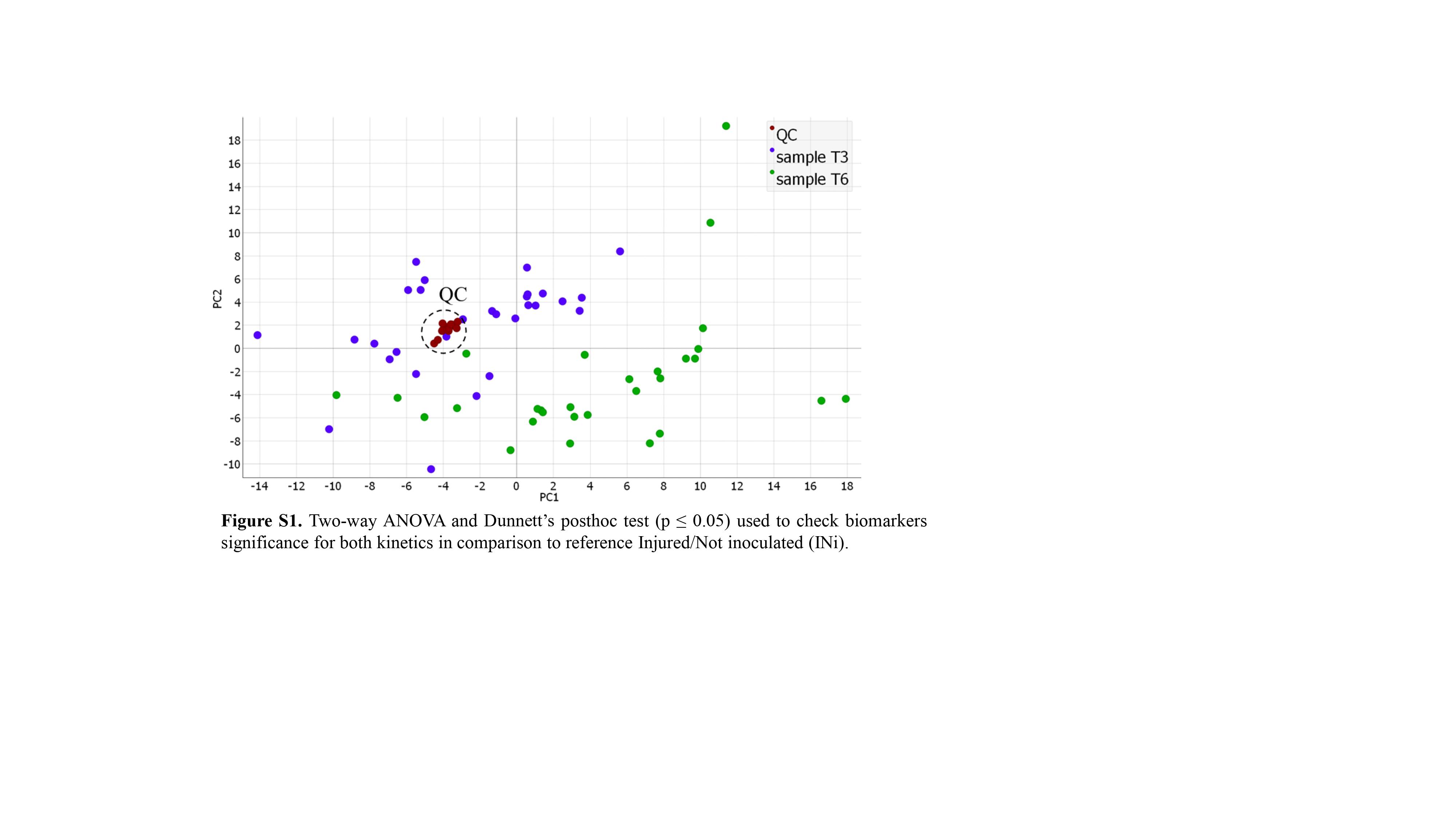

Supplement: Supplementary file 1 [file Image_1.JPEG]

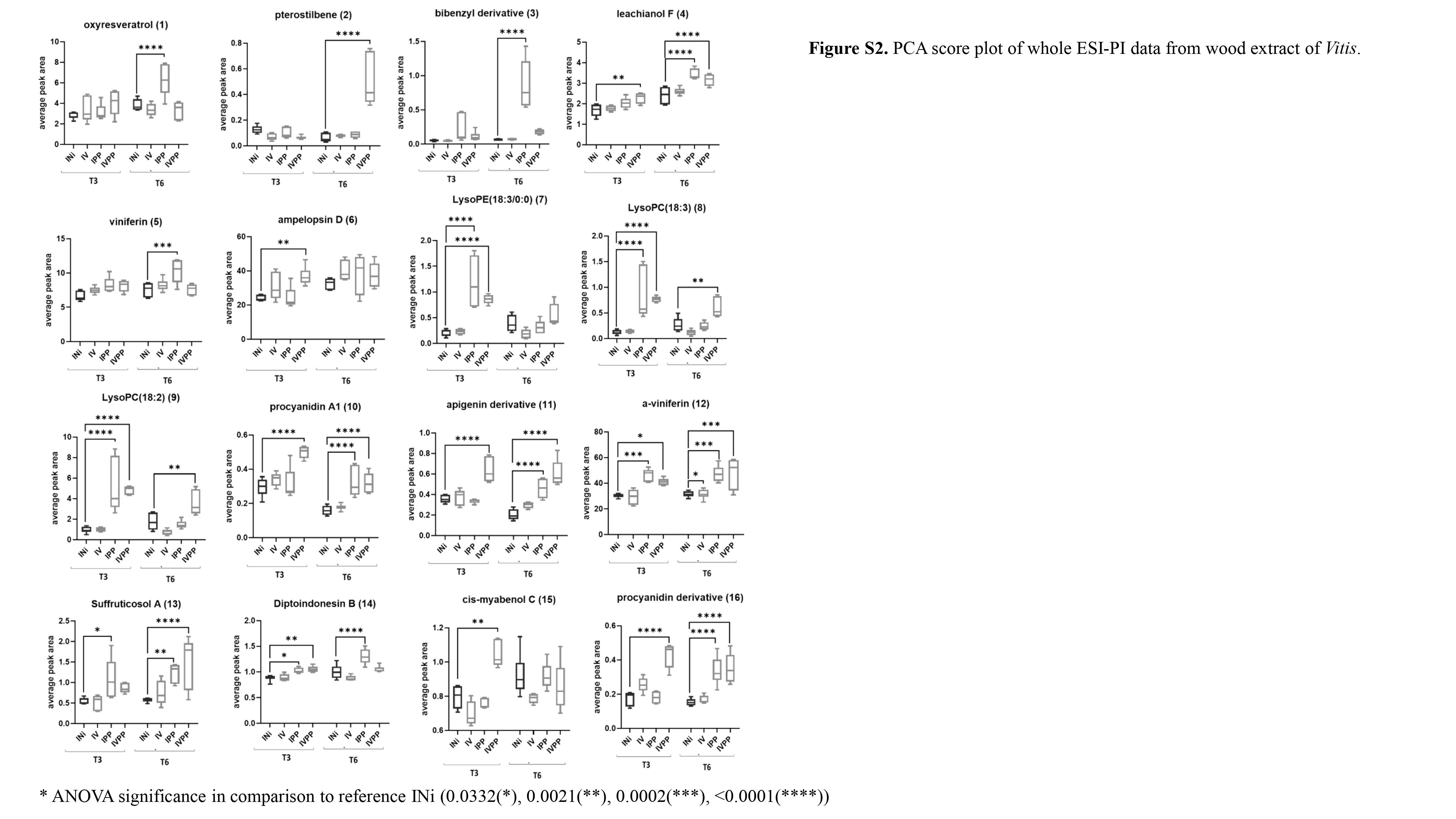

Supplement: Supplementary file 2 [file Image_2.JPEG]

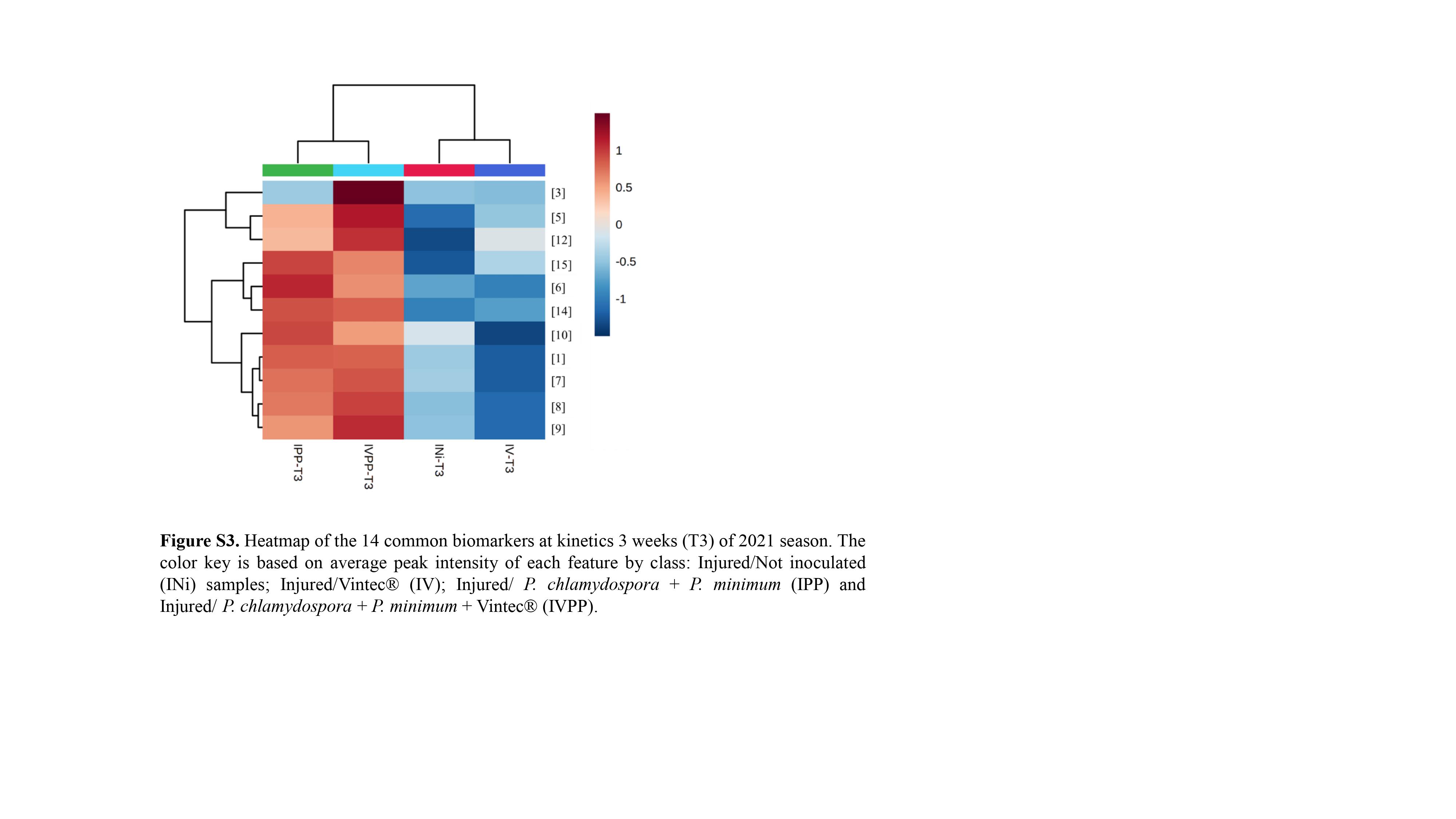

Supplement: Supplementary file 3 [file Image_3.JPEG]

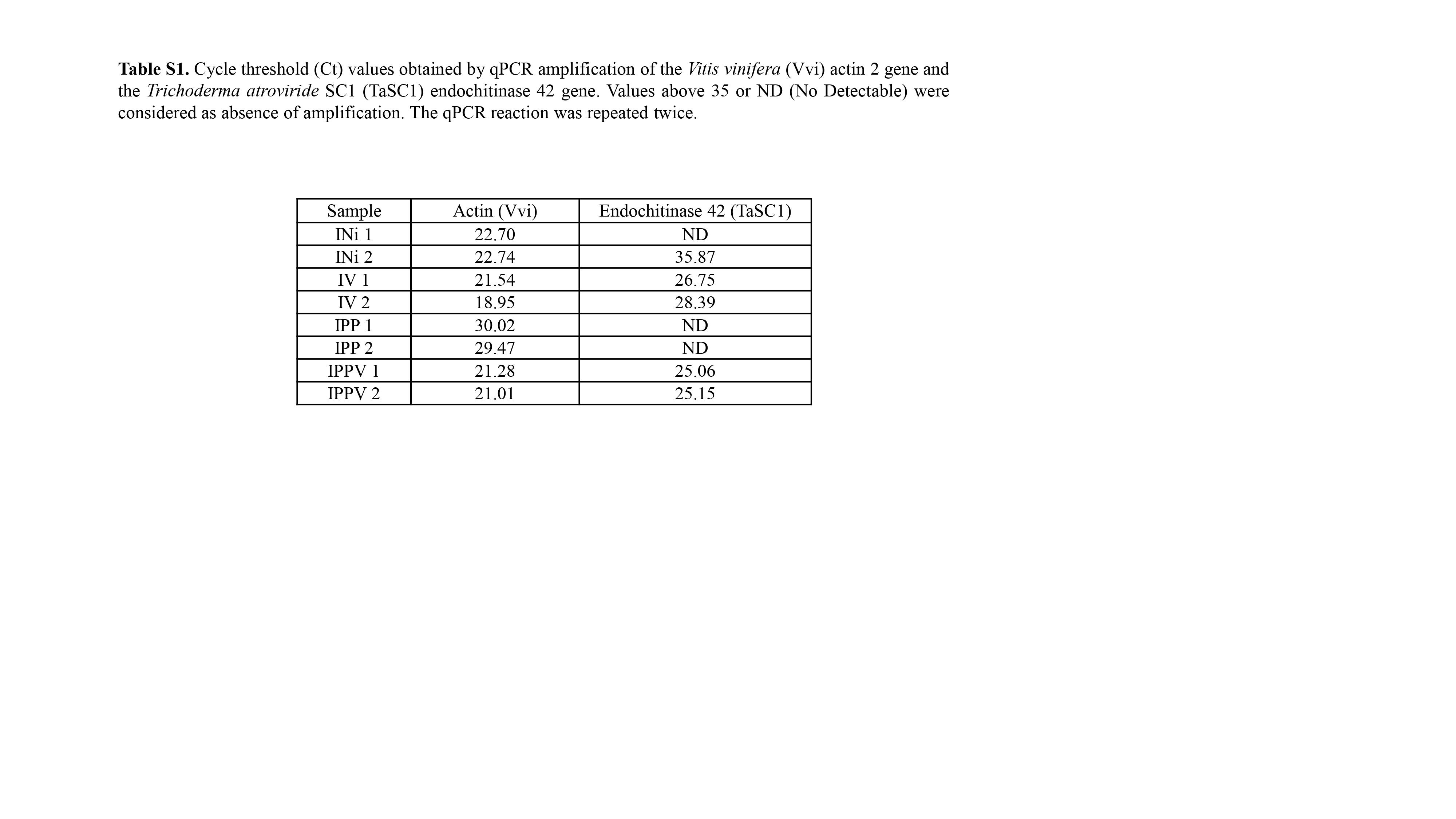

Supplement: Supplementary file 4 [file Image_4.JPEG]

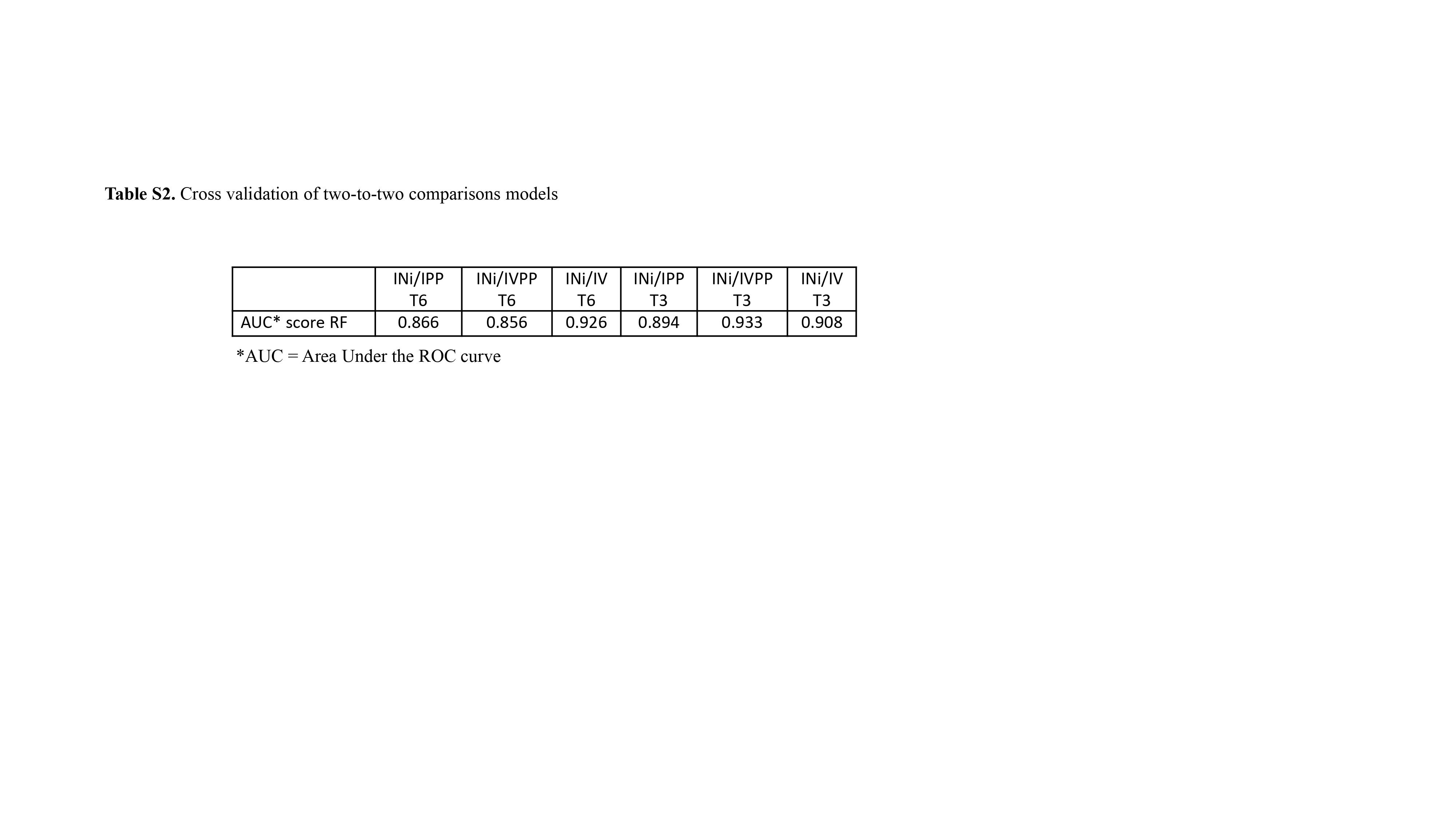

Supplement: Supplementary file 5 [file Image_5.JPEG]
